# *E. coli* bacteremia in comparison to *K. pneumoniae* bacteremia: influence of pathogen species and ESBL production on 7-day mortality

**DOI:** 10.1186/s13756-016-0138-z

**Published:** 2016-10-19

**Authors:** R. Leistner, A. Bloch, P. Gastmeier, F. Schwab

**Affiliations:** 1 Institute of Hygiene and Environmental Medicine, Charité Universitätsmedizin Berlin, Hindenburgdamm 27, 12203 Berlin, Germany; 2 National Reference Center for the Surveillance of Nosocomial Infections, Berlin, Germany; 3 Department of Medical and Financial Controlling, Charité Universitätsmedizin Berlin, Berlin, Germany

**Keywords:** ESBL, Bacteremia, Sepsis, *K. pneumoniae*, *E. coli*, *Enterobacteriaceae*, Mortality

## Abstract

In a previous study, we demonstrated prolonged length of hospital stay in cases of extended-spectrum beta-lactamase (ESBL)-positive *K. pneumoniae* bacteremia compared to bacteremia cases due to *E. coli* (ESBL-positive and –negative) and ESBL-negative *K. pneumoniae*. The overall mortality was significantly higher in bacteremia cases resulting from ESBL-positive pathogens but also in *K. pneumoniae* cases disregarding ESBL-production. In order to examine whether pathogen species rather than multidrug resistance might affect mortality risk, we reanalyzed our dataset that includes 1.851 cases of bacteremia.

## Background

The incidence of infections associated with multidrug-resistant Gram-negative organisms like extended-spectrum beta-lactamase producing Enterobacteriaceae (ESBL-E) is growing in Germany [1]. These infections, according to reports, result in increased mortality and an elevated financial burden for hospitals and health care systems [2]. In 2014, we published the results of a study on the costs of bloodstream infections (BSI) with ESBL-E and length of resulting hospital stays [3]. Our data showed that in particular *K. pneumoniae* was related to prolonged length of stays and increased hospital costs regardless the detection of ESBL production. We furthermore observed a significantly increased overall mortality associated with ESBL-positive pathogens (25.0 % vs. 19.0 %, *p*-value = 0.028). However, this difference in mortality was also observed in *K. pneumoniae* cases compared to *E. coli* cases without considering ESBL-production (25.0 % vs. 18.5 %, *p* = 0.006). This observation posed the question whether the pathogen species or multidrug resistance had a greater influence on the mortality rate.

## Methods

We reanalyzed the dataset to examine risk factors for mortality including pathogen species and ESBL production. All definitions remained the same from the previous publication with the exception of mortality rate. In order to detect mortality rates attributable to the bloodstream infection (BSI-attributable mortality), we focused on the 7-day mortality rate after BSI onset. All other definitions were following the original publication. The median and the interquartile range (IQR) were calculated for continuous parameters, numbers and percentage were calculated for binary parameters. Differences between deceased patients and those who survived were tested using the Wilcoxon rank sum test for continuous variables and the Chi square test for binary variables. A *p*-value <0.05 was considered statistically significant. In the multivariable analysis, Cox-proportional hazard regression analysis was performed to investigate the risk factors for mortality ≤7 days after BSI onset (BSI-attributable mortality). We calculated adjusted hazard ratios (HR) for pathogen species and ESBL-positivity. Model building strategy was step-wise forward with a *p*-value < .05 for including a variable in the model. All parameters with a *p*-value <0.1 in the univariable analysis were considered in the multivariable analysis, and the interaction between pathogen species and ESBL-positivity were included in all multivariable models. Underlying comorbidities were assessed as in the original publication. In order to enable a comparison between the risk factors for BSI-attributable mortality (7-day mortality) and in-hospital mortality, the analysis was repeated for the latter using the same parameters and statistical method. All analyses were performed using SPSS (IBM SPSS statistics, Somer, NY, USA) and SAS (SAS Institute, Cary, NC, USA).

## Results

Overall 8.7 % (*n* = 161) of the patients were deceased within seven days of developing a bloodstream infection whereas *n* = 205 (11.1 %) patients died at a later point in time during their hospital stay (Table 1). In the univariable analysis, patients who died within the 7 days after BSI onset were significantly older compared to the 7-day survivors. However, this was not observed concerning overall in-hospital mortality. With respect to the 7-day mortality, there was no statistical significant difference observed concerning ESBL-production or pathogen species. Furthermore, the following parameter showed significantly increased risk for overall in-hospital mortality but not for 7-day mortality: polymicrobial BSI, male sex, hospital onset, cerebrovascular disease, lung disease and cancer/immunological disease.

**Table 1:** Univariate analysis of 7 days mortality in patients with extended-spectrum beta-lactamase (ESBL)-positive or -negative *E. coli* and *K. pneumoniae* bloodstream infection (during hospitalization)

Parameter	Survived until 7 days after BSI	Died within 7 days after BSI	7-days Mortality	*P*-value	Survived/ discharged after BSI	Died in-hospital after BSI	In-hospital Mortality	*P*-value
	Number (%)	Number (%)			Number (%)	Number (%)		
Patients	**1.690 (100 %)**	**161 (100 %)**	8.7 %		**1485 (100 %)**	**366 (100 %)**	19.8 %	
*K. pneumoniae*	319 (18.9 %)	33 (20.5 %)	9.4 %	0.616	264 (17.8 %)	88 (24.0 %)	25.0 %	**0.006**
*E. coli*	1371 (81.1 %)	128 (79.5 %)	8.5 %		1221 (82.2 %)	278 (76.0 %)	18.5 %	
ESBL-positive	219 (13.0 %)	25 (15.5 %)	10.2 %	0.357	183 (12.3 %)	61 (16.7 %)	25.0 %	**0.028**
Hospital mortality	205 (12.1 %)	161 (100.0 %)	**44.0 %**	**<0.001**	-	366 (100.0 %)	**100.0 %**	n.a.
Polymicrobial	198 (11.7 %)	27 (16.8 %)	12.0 %	0.061*	160 (10.8 %)	65 (17.8 %)	28.9 %	<0.001*
Age (years)^a^	65 (52–73)^a^	67 (57–76)^a^	n.a.	0.019*	65 (52–73)^a^	66 (54–74)^a^	n.a.	0.134*
Male	929 (55.0 %)	93 (57.8 %)	9.1 %	0.496	799 (53.8 %)	223 (60.9 %)	21.8 %	**0.014**
Hospital onset	746 (44.1 %)	84 (52.2 %)	10.1 %	0.050*	612 (41.2 %)	218 (59.6 %)	26.3 %	<0.001*
ICU stay	1.072 (63.4 %)	125 (77.6 %)	10.4 %	<0.001*	896 (60.3 %)	301 (82.2 %)	25.1 %	<0.001*
Charlson comorbidity index^a^	5 (3–8)^a^	8 (6–10)^a^	n.a.	**<0.001**	5 (3–8)^a^	8 (6–10)^a^	n.a.	**<0.001**
Heart disease	275 (16.3 %)	47 (29.2 %)	14.6 %	<0.001*	215 (14.5 %)	107 (29.2 %)	33.2 %	<0.001*
Cerebrovascular disease	243 (14.4 %)	31 (19.3 %)	11.3 %	0.096*	202 (13.6 %)	72 (19.7 %)	26.3 %	0.003*
Neurologic disease	123 (7.3 %)	11 (6.8 %)	8.2 %	0.835	112 (7.5 %)	22 (6.0 %)	16.4 %	0.311
Lung disease	218 (12.9 %)	24 (14.9 %)	9.9 %	0.470	181 (12.2 %)	61 (16.7 %)	25.2 %	**0.023**
Gastrointestinal disease	53 (3.1 %)	12 (7.5 %)	18.5 %	0.004*	36 (2.4 %)	29 (7.9 %)	44.6 %	<0.001*
Rheumatic disease	50 (3.0 %)	2 (1.2 %)	3.8 %	0.208	44 (3.0 %)	8 (2.2 %)	15.4 %	0.420
Liver disease	258 (15.3 %)	53 (32.9 %)	17.0 %	<0.001*	180 (12.1 %)	131 (35.8 %)	42.1 %	<0.001*
Diabetes	422 (25.0 %)	48 (29.8 %)	10.2 %	0.177	366 (24.6 %)	104 (28.4 %)	22.1 %	0.138
Renal disease	711 (42.1 %)	115 (71.4 %)	13.9 %	<0.001*	565 (38.0 %)	261 (71.3 %)	31.6 %	<0.001*
Cancer/immunological disease	672 (39.8 %)	75 (46.6 %)	10.0 %	0.092*	571 (38.5 %)	176 (48.1 %)	23.6 %	0.001*

*BSI* bloodstream infection, *LOS* length of stay, *N.a.* not applicable

* Included in the multivariable Cox-proportional hazard regression

^a^ Continuous parameter displayed as median (interquartile range)

*P*-values <0.05 were considered statistically significant

In the multivariable analysis, independently associated factors with 7-day mortality were renal disease, liver disease, cancer /immunological disease and heart disease (Table 2). No effect was observed concerning neither pathogen species nor ESBL-production. For overall in-hospital mortality, a similar risk profile could be observed concerning underlying diseases. Here, BSI with ESBL-negative *K. pneumoniae* was found to be an independent risk factor for overall in-hospital mortality.

**Table 2:** Multivariable Cox-proportional hazard regression of risk factors for 7-day mortality and hospital mortality after BSI with ESBL-positive or -negative *E. coli* and *K. pneumoniae*

Parameter	Parameter/ category	7-days mortality (stepwise)			In-hospital mortality^a^		
		HR	CI95	*P*-value	HR	CI95	*P*-value
Interaction effect between pathogen species and ESBL-production	*E. coli* ESBL-negative	1 = Reference		0.947	1 = Reference		0.084
	*E. coli* ESBL-positive	1.16	0.70–1.90	0.571	1.22	0.88–1.68	0.228
	*K. pneumoniae* ESBL-negative	0.98	0.64–1.50	0.926	**1.39**	**1.07–1.82**	**0.015**
	*K. pneumoniae* ESBL-positive	1.05	0.49–2.27	0.894	1.01	0.60–1.67	0.983
Comorbidities	Heart disease	**1.55**	**1.09–2.21**	**0.014**	1.23	0.97–1.56	0.087
	Liver disease	**1.96**	**1.40–2.74**	**<0.001**	**1.85**	**1.48–2.30**	**<0.001**
	Renal disease	**2.86**	**1.99–4.10**	**<0.001**	**2.32**	**1.82–2.95**	**<0.001**
	Cancer/ immunological disease	**1.66**	**1.21–2.28**	**0.002**	**1.58**	**1.28–1.95**	**<0.001**

*ESBL* extended-spectrum beta-lactamase, *HR* hazard ratio, *CI95* 95 % confidence interval, *N.s.* not significant

^a^Analyzing the same parameters as in 7-days mortality

*P*-values <0.05 were considered statistically significant

## Discussion

In earlier studies, ESBL-positive pathogens causing infections were associated with increased mortality [2]. However, recent studies have cast doubt on this general assumption and showed a lack of sufficiently controlled studies especially with respect to antimicrobial treatment [4, 5]. Unfortunately, we do not have sufficient data on antimicrobial treatment and are unable to examine this assumption. Especially carbapenem resistance poses a serious threat, as carbapenems are the most important reserve substances for these infections [6]. However, our cohort was comprised of only two cases resulting from carbapenem-resistant organisms.

In the original publication, *K. pneumoniae* cases were associated with a significantly higher comorbidity index (6 vs. 5, *p* = 0.001). *K. pneumoniae* BSIs were more often associated with the detection of other organisms in the same blood culture (24.4 % vs. 9.9 %, *p* ≤ 0.001) and they occurred later than the respective *E. coli* BSIs (hospital day 6 vs. 1, *p* ≤ 0.001). These observations indicate epidemiological and pathophysiological differences between both groups that we do not yet entirely understand.

In the analysis at hand, we observed severe comorbidities, like renal disease, liver disease or cancer to be the only independently associated risk factors for BSI-attributable mortality. In contrast, ESBL-negative *K. pneumoniae* (in addition to comorbidities) was independently associated with in-hospital mortality. We see this as the result of an unmeasured confounder that is associated with increased risk of mortality in the aftermath of survival from a *K. pneumoniae* BSI.

This analysis has limitations. Our data lacks information on antimicrobial therapy, which could provide further information. However, the patients were treated following current sepsis guidelines. The guidelines do not differentiate between *K. pneumoniae* and *E. coli* and therefore render a systematical therapeutic difference unlikely. Data on source of BSI and on the severity of disease (e.g. sepsis, septic shock) were not sufficiently available. Therefore, we could not adjust for the effect of these potential confounders.

## Conclusions

ESBL-E BSI-attributable mortality was influenced neither by pathogen species nor by ESBL-production. However, we observed epidemiological differences between patients infected with *K. pneumoniae* or *E.coli* that lead to an increased in-hospital mortality, not attributable to the BSI episode. Both pathogens are the most commonly found representatives of ESBL-producing bacteria in clinical isolates. Hence, it is of paramount importance to understand the factors that determine the course of infection and the overall outcome. Thus, further well-designed studies of epidemiological and pathophysiological differences between *K. pneumoniae* and *E. coli* -infections are necessary.

## Abbreviations

BSI: Bloodstream infections
*E. coli*: *Escherichia coli*
ESBL-E: Extended-spectrum beta-lactamase producing Enterobacteriaceae
HR: Hazard ratio
IQR: Interquartile range
*K. pneumoniae*: *Klebsiella pneumoniae*
